# CD146/MCAM defines functionality of human bone marrow stromal stem cell populations

**DOI:** 10.1186/s13287-015-0266-z

**Published:** 2016-01-11

**Authors:** Linda Harkness, Walid Zaher, Nicholas Ditzel, Adiba Isa, Moustapha Kassem

**Affiliations:** Department of Endocrinology and Metabolism, Molecular Endocrinology Laboratory (KMEB), Odense University Hospital, University of Southern Denmark, Winslowparken 25.1, 5000 Odense C, Denmark; Danish Stem Cell Center (DanStem), Panum Institute, University of Copenhagen, Blegdamsvej 3B, Copenhagen, 2200 Denmark; Stem Cell Unit, Department of Anatomy, College of Medicine, King Saud University, 4852 Ash Shaikh Hasan Ibn Abdullah Al Ash Shaikh, Riyadh, 11461 Kingdom of Saudi Arabia

**Keywords:** Bone marrow stem cells, CD146/MCAM, Osteogenic differentiation, Bone formation

## Abstract

**Background:**

Identification of surface markers for prospective isolation of functionally homogenous populations of human skeletal (stromal, mesenchymal) stem cells (hMSCs) is highly relevant for cell therapy protocols. Thus, we examined the possible use of CD146 to subtype a heterogeneous hMSC population.

**Methods:**

Using flow cytometry and cell sorting, we isolated two distinct hMSC-CD146^+^ and hMSC-CD146^−^ cell populations from the telomerized human bone marrow-derived stromal cell line (hMSC-TERT). Cells were examined for differences in their size, shape and texture by using high-content analysis and additionally for their ability to differentiate toward osteogenesis *in vitro* and form bone *in vivo*, and their migrational ability *in vivo* and *in vitro* was investigated.

**Results:**

*In vitro*, the two cell populations exhibited similar growth rate and differentiation capacity to osteoblasts and adipocytes on the basis of gene expression and protein production of lineage-specific markers. *In vivo*, hMSC-CD146^+^ and hMSC-CD146^−^ cells formed bone and bone marrow organ when implanted subcutaneously in immune-deficient mice. Bone was enriched in hMSC-CD146^−^ cells (12.6 % versus 8.1 %) and bone marrow elements enriched in implants containing hMSC-CD146^+^ cells (0.5 % versus 0.05 %). hMSC-CD146^+^ cells exhibited greater chemotactic attraction in a transwell migration assay and, when injected intravenously into immune-deficient mice following closed femoral fracture, exhibited wider tissue distribution and significantly increased migration ability as demonstrated by bioluminescence imaging.

**Conclusion:**

Our studies demonstrate that CD146 defines a subpopulation of hMSCs capable of bone formation and *in vivo* trans-endothelial migration and thus represents a population of hMSCs suitable for use in clinical protocols of bone tissue regeneration.

**Electronic supplementary material:**

The online version of this article (doi:10.1186/s13287-015-0266-z) contains supplementary material, which is available to authorized users.

## Background

Human bone marrow (BM) skeletal stem cells (also termed marrow stromal cells or mesenchymal stem cells) (hMSCs) are multipotent stem cells present in the BM stroma and are capable of mesoderm lineage differentiation (e.g., osteoblasts, adipocytes, and chondrocytes) [1]. hMSCs are being tested for treatment in a number of degenerative diseases, including bone [2, 3] and cartilage [4] regeneration. hMSCs isolated and expanded under current culture conditions that rely on plastic adherence are heterogeneous with respect to differentiation. Single-cell cloning of hMSCs revealed that approximately 20–40 % of the cell population are multipotent MSCs and the remaining are probably cells at different stages of differentiation [5, 6]. In addition, Russell et al. reported that only 50 % of clonal BM hMSCs exhibited tri-lineage differentiation (osteoblasts, adipocytes, and chondrocytes) [7]. We recently reported that canonical osteoblastic markers (e.g., alkaline phosphate and osteocalcin) are not predictive for *in vivo* bone-forming capacity of hMSCs or hMSC “stemness” [8] and that there exist, in MSC cultures, cell populations committed to adipocyte or osteoblast lineages [9]. More recently, lineage-tracing studies *in vivo* have corroborated the presence of heterogeneity within the MSC population [10]. This functional heterogeneity of hMSCs limits the clinical use of MSCs in therapy and may explain the varied results obtained from clinical trials [11, 12]. Thus, one of the challenges facing the use of hMSCs in therapy is the identification of prospective markers that predict their *in vivo* functionality.

A number of studies have isolated and characterized distinct populations of BM hMSCs by using a number of surface markers (e.g., Stro-1 and CD105 [13], CD271 [14], and CD56 [15, 16], and alkaline phosphatase (ALP) [17]). Although these markers enrich for an hMSC population with trilineage differentiation and colony-forming abilities, the isolated cells were still heterogeneous with respect to differentiation potential.

Cluster of differentiation 146 (CD146), also known as melanoma cell adhesion molecule (MCAM, MelCAM) or cell surface glycoprotein Muc18, was originally identified as an endothelial cell marker with a role in cell-matrix interaction and angiogenesis. CD146 defines the self-renewing hMSC population located in perivascular space in BM [18]. Additionally, CD146 expression has been reported to be higher in hMSC multipotent clones compared with hMSC unipotent clones [7] and to be correlated with osteoblastic differentiation potential [18, 19]. Conversely, Tormin et al. [14] reported that multipotent hMSCs are present in both the CD146^−^ and CD146^+^ populations and that these populations exist within two different niches *in vivo*: endosteal and perivascular. Thus, differences and similarities of the CD146^−^ and CD146^+^ populations of hMSCs have not been totally clarified.

We have previously established a human telomerase-overexpressing hMSC line (termed hMSC-TERT [20]) that maintains “stemness” characteristics of hMSCs in spite of extensive *in vitro* proliferation. hMSC-TERT exhibit a mixed expression of CD146 and thus provided us with the opportunity to characterize, in a prospective fashion, the phenotype of hMSCs defined by CD146 expression. Here, we compare the biological characteristics of CD146^+^ and CD146^−^ cell populations by employing *in vitro* and *in vivo* assays.

## Methods

### Cell cultures

We employed the parental telomerised cell line hMSC-TERT (subclone hMSC-TERT4) described previously [20]. To visualize the cells when implanted *in vivo*, we created a sub-line from hMSC-TERT4, overexpressing firefly luciferase (LUC2) and termed hMSC-LUC2.

### Stable retroviral transfection using the firefly luciferase gene LUC2

The LUC2 gene was polymerase chain reaction (PCR)-amplified from the pGL4.10 plasmid by using Phusion Hot Start High-Fidelity DNA Polymerase (Thermo Fisher Scientific, Waltham, MA, USA) in accordance with the instructions of the manufacturer. The primers sequences were as follows: forward primer: CATCAGCCAGCCCACCGTCG and reverse primer: CGGTCGAAGCTCTCGGGCAC.

The PCR products were purified with the SV gel and PCR clean-up system (Promega, Nacka, Sweden) and ligated into the retroviral vector pBABEpuro, which was pre-digested with the SnaBI (blunt) restriction enzyme. The plasmid was then transformed into DH5α cells. Positive clones were identified by purifying the vector by using a Wizard^®^ Plus SV Minipreps DNA Purification System (Promega) in accordance with the instructions of the manufacturer. After purification, the plasmid was linearized and analyzed on a 0.5 % agarose gel for 90 min at 70 V. Finally, the inserts were confirmed by sequencing.

### Virus generation and transfection

Phoenix A packaging cells (70–80 % confluent), cultured in six-well plates, were transfected with the pBABE-LUC2 construct (3 μg/well) by using the FuGENE 6 (Roche, Hvidovre, Denmark) method in accordance with the instructions of the manufacturer. The supernatants, from 25 cm^2^ of Phoenix A packaging cells containing virus particles, were collected 24 and 48 h after transfection, filtered with a 0.45-μm filter, diluted 1:1 with the culture medium, and added to the hMSC-TERT cell line, in a 25-cm^2^ flask supplemented with 6 μg/ml Polybrene for infection. Twenty-four hours after the second round of infection, 3 μg/ml puromycin was added for selection, and the selection pressure was maintained until all non-transfected control cells were eradicated. The puromycin-resistant cells were expanded and maintained in medium supplemented with 0.2 μg/ml puromycin. An estimated 500,000 cells initially survived the selection to make the respective cell line (termed hMSC-LUC2).

### CD146 cell sorting

The hMSC-LUC2, was used for all experiments. hMSC-LUC2 cells were sorted on a FACSdiva (BD Biosciences, Brøndby, Denmark) into positive (hMSC-CD146^+^) and negative (hMSC-CD146^–^) cell populations using incubation with a PE pre-conjugated CD146 antibody (BD Pharmingen, Brøndby, Denmark). Briefly, hMSC-LUC2 cells were trypsinized to a single-cell suspension, washed in phosphate-buffered saline (PBS) with 0.5 % bovine serum albumin (BSA), and incubated with CD146 antibody diluted in PBS with 0.5 % BSA for 30 min on ice. Cells were then sorted into distinct populations on a FACSAria cell sorter (BD Biosciences) and re-cultured for future *in vitro* and *in vivo* experiments. Cell expansion was performed in basal media (minimum essential medium) (Invitrogen, Taastrup, Denmark with 10 % fetal bovine serum (FBS); PAA, Pasching, Austria).

### Cell proliferation

Cell proliferation was monitored by determining the number of population doublings by using the formula: logN/log2, where N is the cell number of the confluent monolayer divided by the initial number of seeded cells.

### Cell differentiation

For osteoblast differentiation, the cells were cultured in osteoblastic induction media (OIM) comprised of basal media supplemented with 10 mM β-glycerophosphate (Calbiochem-Merck, Darmstadt, Germany), 50 μg/ml L-ascorbic acid-2-phosphate (Wako Chemicals GmbH, Neuss, Germany), 10 nM dexamethasone (Sigma-Aldrich, Brøndby, Denmark), and 10 nM calcitriol (1.25-dihydroxy vitamin-D_3_ (1,25 (OH)_2_D_3_) kindly provided by Leo Pharma, Ballerup, Denmark). For adipocyte differentiation, the cells were cultured in adipocytic induction media (AIM) containing basal media supplemented with 10 % horse serum (Sigma-Aldrich), 100 nM dexamethasone (Sigma-Aldrich), 500 μM 1-methyl-3-isobutylxanthine (IBMX) (Sigma-Aldrich), 1 μM Rosiglitazone (BRL49653; Cayman Chemical, Ann Arbor, MI, USA), and 5 μg/ml insulin (Sigma-Aldrich). Samples undergoing induction were collected at days 5, 10, and 15. Three independent experiments were performed for each differentiation assay.

### Flow cytometry

Flow cytometry was performed by using a FACScan (BD Biosciences). To confirm the profile of either hMSC-TERT versus hMSC-LUC2 or hMSC-CD146^+^ and hMSC-CD146^–^ populations, cells were trypsinized to a single-cell suspension, washed in PBS + 0.5 % BSA, and incubated with an antibody (in PBS + 0.5 % BSA) for 30 min on ice. After incubation, excess antibody was washed out by using PBS and cells analyzed on the FACSCalibur (BD Biosciences) flow cytometer and data analyzed by using WinMDI (The Scripps Institute, Flow Cytometry Core Facility). Sorted and unsorted cell populations were profiled using a number of known MSC pre-conjugated fluorescence-activated cell sorting (FACS) markers: CD14-FITC, CD34-PE, CD44-PE, CD63-FITC, CD73-PE, and CD146-PE (all BD Pharmingen) and CD105-APC (eBioscience, Hatfield, UK).

### Alkaline phosphatase activity and cell viability

Cell viability was measured by using CellTiter-Blue Cell Viability assay in accordance with the instructions of the manufacturer (Promega). ALP activity was determined by using a 1 mg/ml solution of P-nitrophenylphosphate (Sigma-Aldrich) in 50 mM NaHCO_3_ with 1 mM MgCL_2_, pH 9.6, at 37 °C for 20 min; activity was then stopped with 3 M NaOH. Plates were read by using a FLUOstar Omega plate reader (BMG Laboratories, Ramcon A/S, Birkerod, Denmark), and ALP activity was corrected for cell number.

### Cytochemical staining

Cells undergoing osteoblastic or adipocytic induction were stained at days 5, 10, and 15 for ALP and alizarin red or Oil red O (ORO), respectively, as previously described [21]. Elution of ORO staining was performed by using 100 μl of isopropanol for 10 min at room temperature; 60 μl was then removed to a 96-well plate and read on a FLUOstar Omega plate reader set at 500 nm emission wavelength.

### Cell morphology analysis by high-content imaging

hMSC-CD146^+^ and hMSC-CD146^−^ cells were seeded in 96-cell carrier well plates (PerkinElmer, Waltham, MA, USA) and cultured in standard medium. The adherent cells were washed with PBS^−/−^, fixed in 4 % formaldehyde for 10 min at room temperature, and washed three times with PBS. Cells were stained for alpha tubulin, Phalloidin-TRITC, and Hoechst 33342 (all Sigma-Aldrich) by using Alexafluor 488 (Thermo Fisher Scientific) as the secondary antibody for alpha tubulin. Plates were scanned in the Operetta (PerkinElmer) and cells (>1000 per cell line) analyzed for cell size and shape and changes in microtubule (F-actin) thickness using the Harmony software (PerkinElmer) and texture analysis using SER (saddle, edge, ridge) features to measure changes in structure within the cell. Both the ‘Valley’ and ‘Ridge’ patterns from the SER features were selected by using a 2px width to assess spaces or ridge height of the F-actin fibres.

### Boyden chamber transwell migration assay

The migration ability of sorted cells was measured in a 48-well micro-chemotaxis Boyden chamber-based cell migration assay (8-μm pore polycarbonate membrane; Neuro Probe, Inc., Gaithersburg, MD, USA). The membrane was coated for 1 h at 37 °C in migration media—low glucose Dulbecco’s modified Eagle’s medium (Invitrogen), with 0.2 % FBS (Sigma-Aldrich)—supplemented with 5 μg/ml Fibronectin and 10 μg/ml rat-tail collagen I. The cells were cultured in migration media for 24 h prior to migration initiation. The chemotaxis and control media (27 μl) were placed in the lower chamber. The chamber was covered by the coated membrane and fixed by the upper chamber. Fifty-microliter sorted cells (2.5 × 10^5^ cells/ml migration media) were loaded into the upper chamber wells and incubated for 4 h at 37 °C. Non-migrated cells on the upper side of the membrane were scraped and rinsed with cold PBS. Migrated cells on the lower side of the membrane were fixed and stained with a hemacolor^®^ staining kit (Merck) and imaged on an inverted microscope (×20 magnification). Images were captured of the entire wells, and cells were counted by using the ImageJ program. Results were expressed as mean number of cells for six technical replicates (n = 2 independent experiments).

### Gene expression analysis

Total RNA was extracted from samples collected at days 0, 5, 10, and 15 of induction from unsorted and sorted populations by using TRIzol (Invitrogen) in accordance with the instructions of the manufacturer. cDNA was synthesized by using a revertAid H minus first-strand cDNA synthesis kit (Fermentas, St Leon-Rot, Germany). Reverse transcription-polymerase chain reaction (RT-PCR) was performed on an ABI StepOne™ Real-Time PCR machine (Life Technologies/Applied Biosystems), and data analyzed by using Excel (Microsoft Corporation, Redmond, WA, USA). Ten micrograms of cDNA was used in each reaction in combination with 2× Fast SYBR green master mix (Applied Biosystems). Primers were purchased from DNA Technology A/S (Risskov, Denmark) or Eurofins (Ebersberg, Germany) and included Collagen 1a1 (*COL1a1*), *RUNX2*, alkaline phosphatase (*ALPL*), osteocalcin (*BGLAP*), osteonectin (*SPARC*), osteopontin (*OPN*), the long and short forms of CD146 (*lgCD146*, *shCD146*), *CD146*, elastin (*ELN*), decorin (*DCN*), and biglycan (*BGN*) for osteogenic differentiation and adipocyte-specific lipid binding protein 2 (*aP2*), *PPARγ2*, lipoprotein lipase (*LPL*), adiponectin (*ADIPOQ*), and *C/CEBPα* for adipogenic differentiation. Cycle threshold (CT) values were normalized against β-actin and analysis was performed by using the comparative CT method. Statistical analysis to detect differences between gene expression levels was carried out by using Student’s *t* test. Primers used in these studies can be found in Additional file 1: Table S1.

### *In vivo* heterotopic bone formation assay

The cells were trypsinized to a single-cell suspension and seeded onto 40 mg Triosite (HA/TCP, 0.5- to 1-mm granules; Biomatlante/Zimmer, Vigneux de Bretagne, France) and kept overnight at 37 °C, 5 % CO_2_. HA/TCP granules in combination with cells were then implanted subcutaneously (four implants per cell line) in the dorso-lateral area of immune-compromised (NOD.CB17-*Prkdc*^*scid*^/J) mice (The Jackson Laboratory, Bar Harbor, ME, USA) for 8 weeks. Ethical approval for subcutaneous implantations was granted by the Danish National Animal Experiment Inspectorate (2012-DY-2934-00006). The implants were removed, fixed in formalin, decalcified using formic acid for 3 days or 0.4 M ethylenediaminetetraacetic acid (EDTA) for 12 days, embedded, and sectioned. Four serial sections were cut at three different tissue depths with 100 μm distance between each group. Staining was performed with hematoxylin and eosin (three sections) or human-specific Vimentin (#RM-9120, Clone SP20; Thermo Fisher Scientific: one section). Additional staining was performed by using tartrate-resistant acid phosphate (TRAP) staining on implants which underwent 0.4 M EDTA decalcification. In brief, 50 ml acetate buffer pH 5.2 with 115 mg sodium tartrate and 70 mg Fast Red Salt TR (Sigma-Aldrich, F2768) was mixed with 70 mg napthol AS-TR phosphate (Sigma-Aldrich, N6125) dissolved in 250 μl N-N-dimethylformamide to produce the staining solution. The slides with tissue sections were deparaffinized in xylene and processed through increasing alcohol concentrations before being incubated in the staining solution for 2 h at 37 °C. Sections were briefly counterstained in Mayer’s hematoxylin before rinsing in tap water and mounting under coverslips with an aqueous mounting medium.

### Quantification of heterotopic bone formation and bone marrow

Mosaic images of serial sections were obtained by using Surveyor (Objective Imaging, Cambridge, UK). Using the free-hand contouring tool from PhotoShop (Adobe, CS4), the tissue area of each implant was calculated. Using the Magic wand-tool, the bone or BM areas were highlighted and the areas calculated; data were then calculated as a percentage of bone or BM/tissue area of the implant (nine sections per implant).

### *In vivo* migration assay

A closed fracture was created in 10 immunodeficient (NOD.CB17-*Prkdc*^*scid*^/J; The Jackson Laboratory) 8-week-old, sedated, female mice (100 mg/kg ketamine hydrochloride and 5 mg/kg xylazine hydrochloride) as described by Bonnarens and Einhorn [22]. Ethical approval for femoral fracture was granted by the Danish National Animal Experiment Inspectorate (2012-15-2934-00559). Fractures were created by using a guillotine apparatus on the right hind limb following insertion of a 0.5-mm needle. Fracture and pinning were confirmed by x-ray (Faxitron MX-20). Three hours after fracture, single-cell suspended hMSC-CD146^+^ or hMSC-CD146^–^ cells were tail vein-injected at 1 × 10^6^ cells per 100 μl of PBS/injection into five mice per cell line and bioluminescence imaged at 30 min post-injection (d0), d1, d3, and d6.

### Bioluminescence imaging

The Xenogen IVIS Spectrum (PerkinElmer) was used to obtain images of bioluminescence. Ten to 15 minutes prior to imaging, immunodeficient mice, tail vein-injected with either hMSC-CD146^+^ or hMSC-CD146^−^ cells, were injected intraperitoneally with D-luciferin (PerkinElmer, 150 mg/kg body weight in sterile PBS without Mg^2+^ and Ca^2+^) and then sedated with 2.5 % isoflurane in oxygen. Images were obtained with field-of-view 13.2 cm, binning factor 8, F/Stop 1, and automatic exposure time by using Xenogen Living Image™ software (version 2.11) overlay (PerkinElmer). Radiance was plotted by using calibrated measurements of photon emission and displayed in units of photons/second/cm^2^/steradian.

### Statistical analysis

Three independent *in vitro* experiments were performed, and data were presented as mean ± standard deviation. Statistical analysis were performed by using a one-way analysis of variance where group size was 3 or more, and for group sizes of 2, Student’s *t* test was applied (*P < 0.05, **P < 0.005 or *P* < 0.01, ***P < 0.0001).

## Results

### Characterization of hMSC-LUC2 cell line

hTERT-LUC2 exhibited cell morphology (Additional file 2: Figure S1A) and CD marker expression profile (Additional file 2: Figure S1C) similar to hMSC-TERT. However, hMSC-LUC2 demonstrated lowered proliferation rate compared with hMSC-TERT: population doubling per day was 0.45 ± 0.06 versus 0.58 ± 0.04, respectively (*P* < 0.005) (Additional file 2: Figure S1B). *Ex vivo* differentiation studies demonstrated that hTERT-LUC2 maintained their osteoblastic (Additional file 3: Figure S2A) and adipocytic (Additional file 3: Figure S2B) differentiation capacities. RT-PCR data demonstrated similar gene expression profiles between the hMSC-TERT and hTERT-LUC2. Similar to hMSC-TERT, hMSC-LUC2 formed heterotopic bone and BM when implanted subcutaneously in immune-deficient mice (Additional file 4: Figure S3).

### Isolation and phenotypic characterization of hMSC-CD146^+^ and hMSC-CD146^–^ cells

**Fig. 1:**
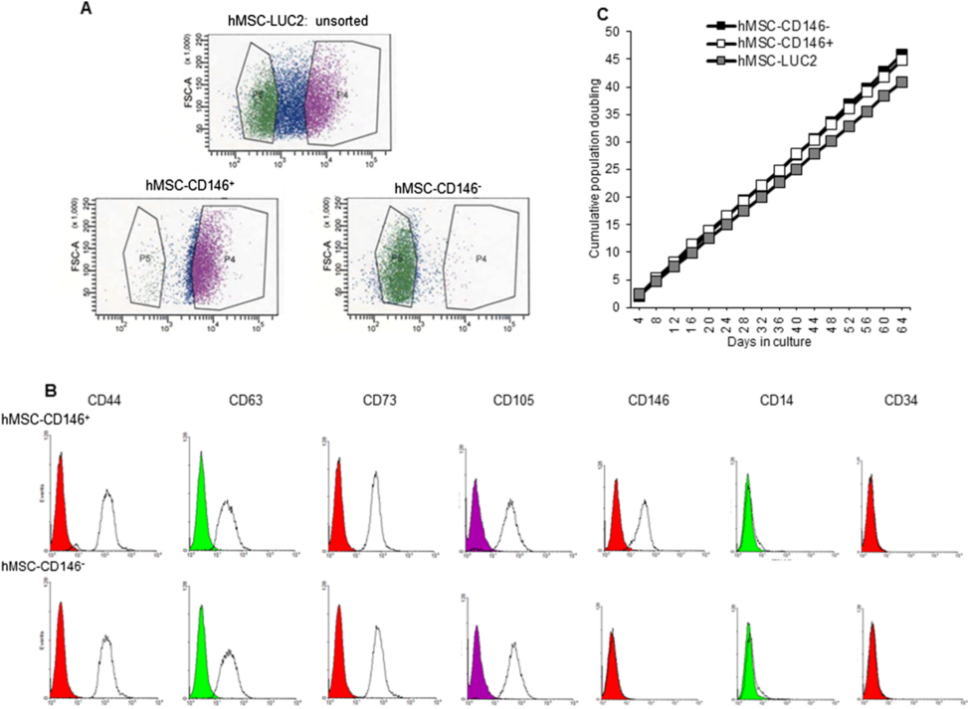
Flow cytometric cell sorting and cell characterization. **a** Details from FacsDIVA sorting demonstrating the overall distribution of CD146 in hMSC-TERT and the gated CD146^+^ (P4) and CD146^−^ (P5) populations. **b** Flow cytometric validation of the hMSC-CD146^+^ and hMSC-CD146^−^ populations and expression of canonical mesenchymal stem cell (MSC) markers: CD44, CD63, CD73, CD105, CD14, CD34, and CD146. **c** Analysis of population doublings demonstrating no significant difference between the different cell populations. *FSC-A* forward scatter-A, *hMSC* human mesenchymal stem cell

hMSC-LUC2 cells (18 × 10^6^) were sorted on the basis of the expression of CD146. Two distinct areas were gated (P4: hMSC-CD146^+^ and P5: hMSC-CD146^–^; Fig. 1a), and cells remaining within the intermediary area (40 % of the total population) were discarded. After sorting of the hMSC-CD146^+^ cells, 75.5 % were gated within P4 and 89.1 % of cells were gated within P5 (hMSC-CD146^–^) (Fig. 1a). Immediately after cell sorting and for subsequent passages when cells were used for experimental purposes, flow cytometry was performed to confirm the CD146 status in both cell populations. The cells maintained their CD146^+^ or CD146^−^ status for six passages. In addition, the MSC identity of the cells was maintained as shown by the expression of a selected number of CD markers by FACS analysis: CD14 and CD34 (both negative) and CD44, CD63, CD73, and CD105 (all positive). We observed similar expression levels of these markers in hMSC-CD146^+^ and hMSC-CD146^–^ (Fig. 1b). Growth rate was identical between hMSC-LUC2, hMSC-CD146^+^, and hMSC-CD146^–^ (Fig. 1c).

### Quantitative cell morphology using high-content imaging

**Fig. 2:**
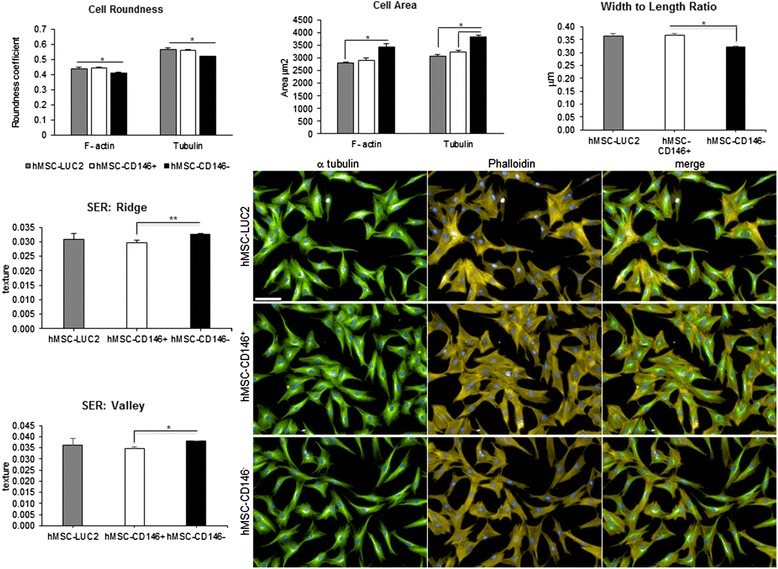
Morphological and texture studies using high-content single-cell imaging. Fluorescent staining of hMSC-TERT, hMSC-CD146^+^, and hMSC-CD146^−^ (n = 3) demonstrates morphological changes assessed by tubulin and F-actin staining. Significant changes are demonstrated in cell roundness, area, and width-to-length ratio (*P < 0.05). Changes in F-actin are demonstrated by analysis of depth and height of ridges of the actin fibres. *P < 0.05. Scale bar = 100 μm. *hMSC* human mesenchymal stem cell, *SER* saddle, edge, ridge

hMSC-CD146^+^ and hMSC-CD146^–^ were examined for their differences in cell morphology and texture using high-content imaging analysis. Significant differences in cell area, roundness, and width-to-length ratio were observed between hMSC-CD146^+^ and hMSC-CD146^−^ (Fig. 2). hMSC-CD146^+^ were rounder than hMSC-CD146^−^ cells and demonstrated a smaller cytoplasmic area. Analysis of the F-actin filaments (stained with Phalloidin) using SER texture analysis demonstrated that the hMSC-CD146^−^ cells contained significantly larger fibres than hMSC-CD146^+^ (*P* < 0.05; Fig. 2).

### Osteoblast differentiation of hMSC-CD146^+^ and hMSC-CD146^–^

**Fig. 3:**
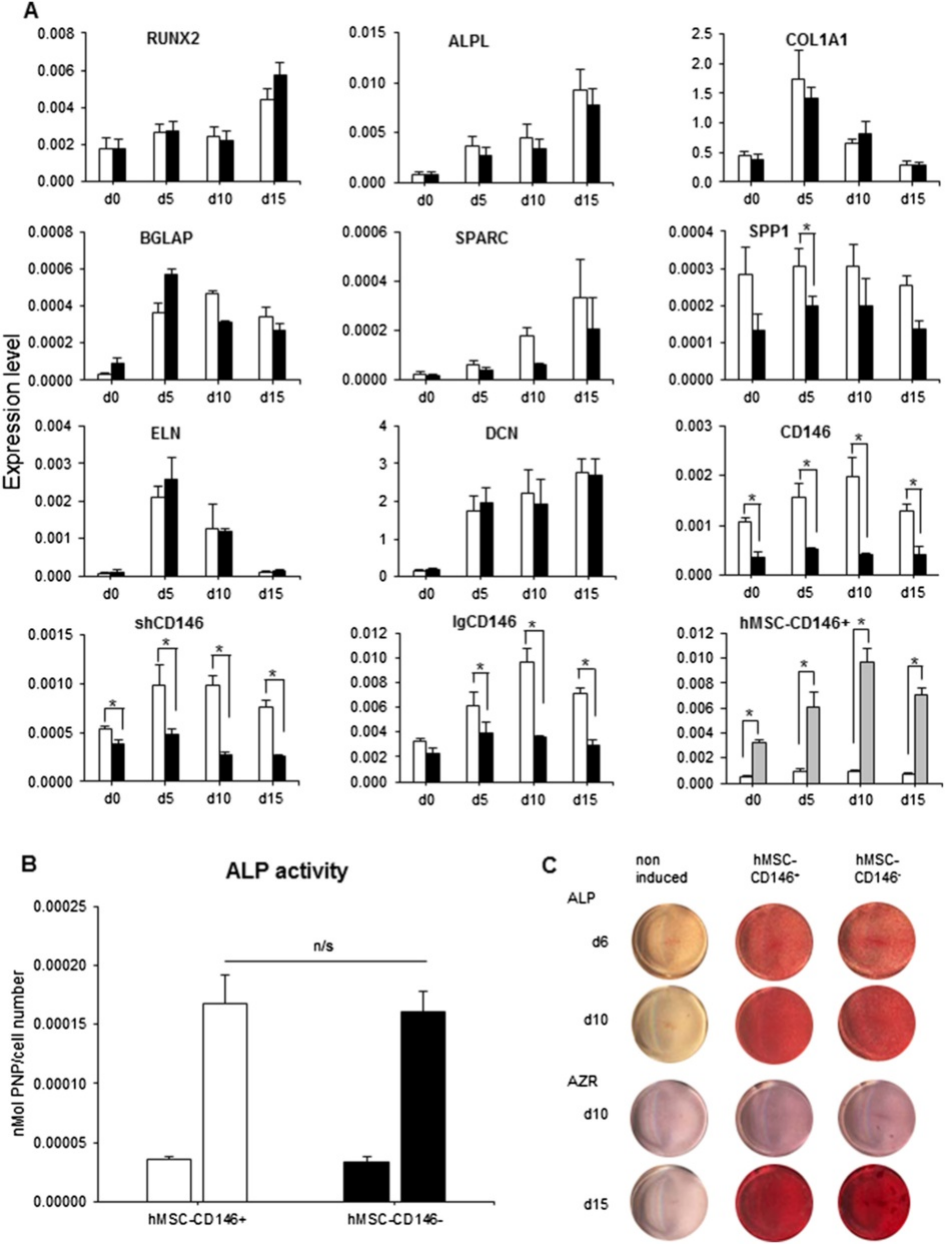
Characterization of sorted CD146 populations undergoing osteoblast (OB) differentiation. **a** Reverse transcription-polymerase chain reaction data (mean ± standard error of the mean, n = 3 independent experiments) analyzed from sorted population at day (d) 0, 5, 10, and 15. *P < 0.05. sh = short form and lg = long form of CD146. **b** Alkaline phosphatase (ALP) activity/cell viability at d6 of osteoblastic differentiation. **c** ALP staining at d6 and d10 of OB differentiation. Alizarin red (AZR) staining at d10 and d15 of OB differentiation. n = 3 independent experiments. white box, hMSC-CD146^+^; black box, hMSC-CD146^−^; gray box, lgCD146^+^

No consistent significant differences between gene expression levels in hMSC-CD146^+^ and hMSC-CD146^–^ were observed (Fig. 3a). Similarly, no differences were observed between hMSC-CD146^+^ and hMSC-CD146^–^ in ALP activity (Fig. 2b) or ALP cytochemical staining (Fig. 3c) or in their ability to form mineralized matrix as visualized by alizarin red staining (Fig. 3c). The phenotype of hMSC-CD146^+^ and hMSC-CD146^–^ was stable during osteoblast differentiation as demonstrated by gene expression levels of CD146 isoforms (Fig. 3a).

CD146 was previously reported to have two isoforms—short and long, generated by alternative splicing of the cytoplasmic region—which probably mediate different functions [23]. We found that the long form of CD146 (lgCD146), but not the short form (shCD146), expression levels were upregulated during OB differentiation (*P* < 0.05) (Fig. 3a).

### Adipocytic differentiation of hMSC-CD146^+^ and hMSC-CD146^–^

Both hMSC-CD146^+^ and hMSC-CD146^–^ exhibited similar differentiation capacity to adipocytes as revealed by comparable gene expression levels of adipocytic differentiation markers: peroxisome proliferator-activated receptor gamma 2 (*PPARγ2*), lipoprotein Lipase (*LPL*), fatty acid binding protein 4 (*FABP4/aP2*), CCAAT/enhancer binding protein alpha (*C/EBPα*), adiponectin (*ADIPOQ*) as well as oil red O staining of mature adipocytes and quantitative measurements of eluted oil red O as an indicator of the number of mature, lipid filled adipocytes (Additional file 5: Figure S4).

### *In vivo* heterotopic bone formation

**Fig. 4:**
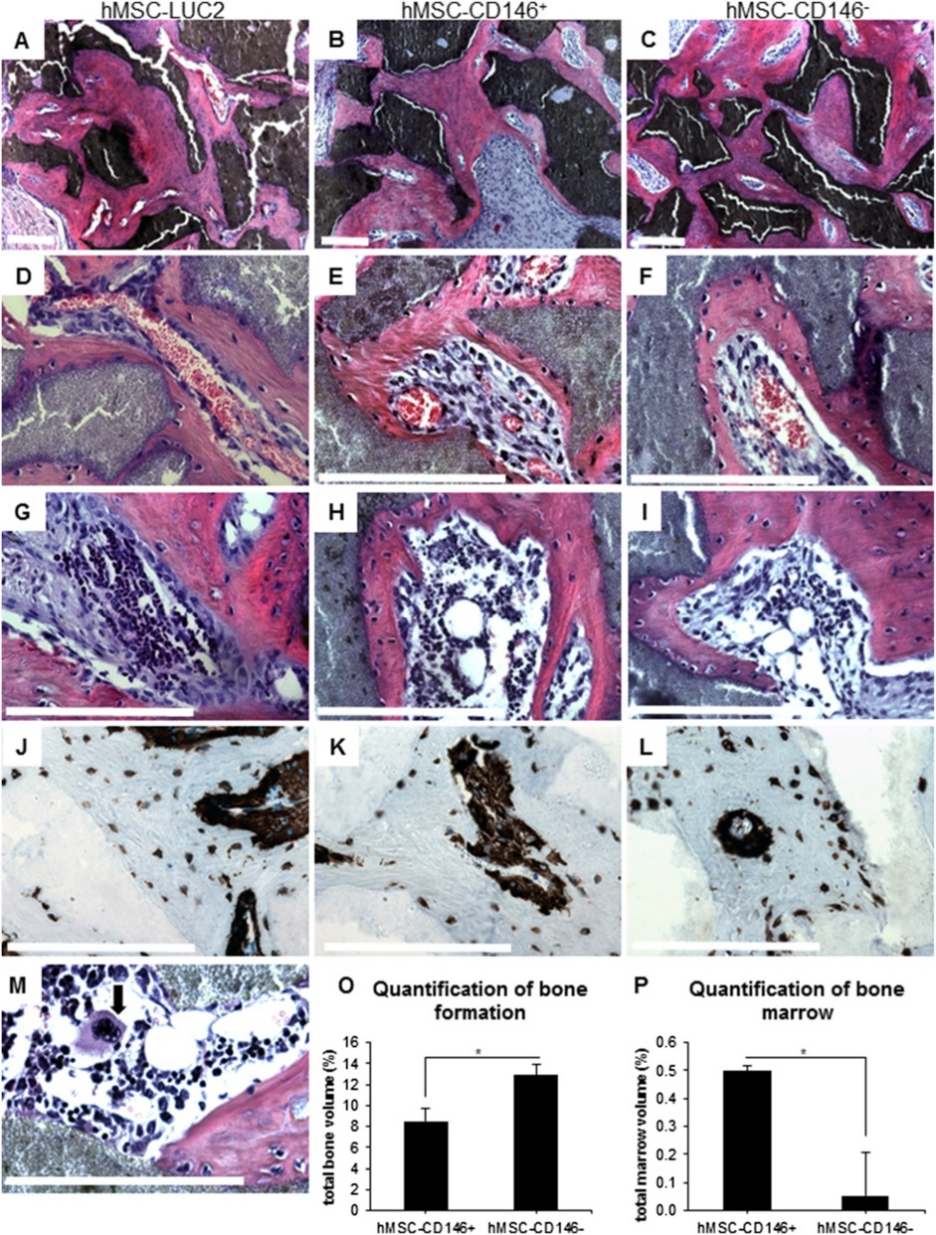
*In vivo* heterotopic bone formation. hMSC-LUC2 (**a**, **d**, **g**, **j**, **m**), hMSC-CD146^+^ (**b**, **e**, **h**, **k**), and hMSC-CD146^−^ (**c**, **f**, **i**, **l**) were implanted subcutaneously (n = 4) mixed with hydroxyapatite tricalcium phosphate/Triosite (HA/TCP) into immune-compromised mice for 8 weeks. Analysis was performed on three serial sections at three depths with 100 μm between each depth. Hematoxylin and eosin (H&E) staining of bone formation demonstrating distribution of bone within the implants (**a**-**i**, **m**). **d**-**f** Blood vessel formation within the implants. **g**-**i** Establishment of bone marrow within the implants. **j**-**l** Human-specific vimentin staining of developing bone demonstrating the human origin. **o**, **p** Quantification of total bone or bone marrow volume. Scale bar on all H&E and vimentin staining = 100 μm

We examined the ability of hMSC-CD146^+^ and hMSC-CD146^–^ populations to form heterotopic bone when implanted subcutaneously in immune-deficient mice together with HA/TCP. The CD146 status of the cells was confirmed by flow cytometry prior to implantation (Additional file 6: Figure S5). Bone formation was demonstrated in both hMSC-CD146^+^ and hMSC-CD146^–^ (Fig. 4). Formation of BM within the implants was identified on the basis of the presence of large sinusoids (Fig. 4d-f) and BM elements (Fig. 4g-i, m). Quantification of bone formation demonstrated a significant difference between hMSC-CD146^−^ (12.6 ± 1.5) and hMSC-CD146^+^ (8.1 ± 1.0) (*P* < 0.05) (Fig. 4o). In contrast, significantly (*P* < 0.01) increased amounts of BM were identified in hMSC-CD146^+^ (0.5 % ± 0.2 %) as compared with hMSC-CD146^–^ (0.05 ± 0.02) (Fig. 4p). Scans of whole implants demonstrating differences in bone formation between hMSC-CD146^+^ and hMSC-CD146^−^ can be found in Additional file 7: Figure S6. Specificity of the vimentin staining was demonstrated by using murine MSCs implanted under similar conditions to hMSCs (Additional file 8: Figure S7). Bone formed in hMSC implants was of human origin as evidenced by positive staining for a human-specific vimentin (Fig. 4j-l).

### *In vitro* and *in vivo* chemotaxis assay

**Fig. 5:**
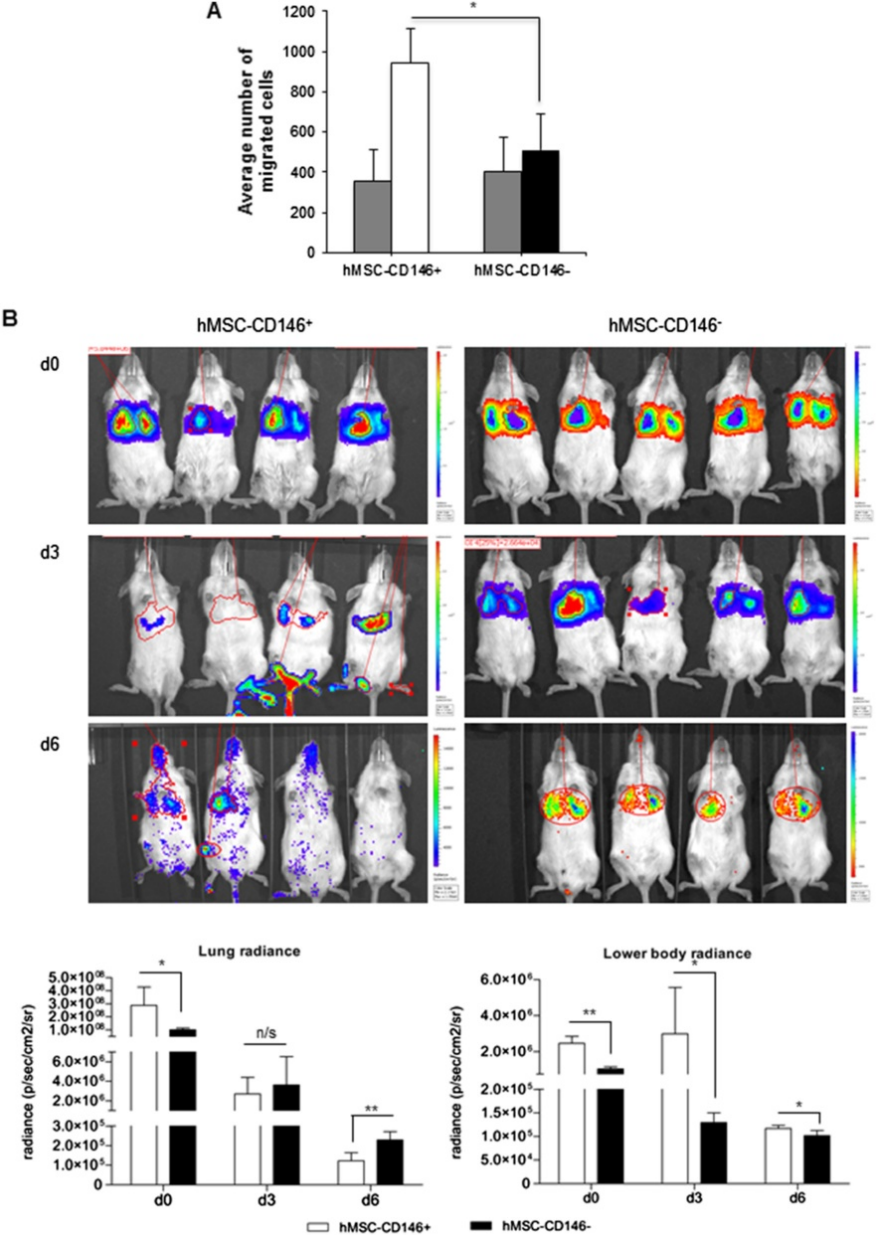
Functional assessment of migrational ability of CD146 sorted human mesenchymal stem cell (hMSC) populations. **a** Analysis of *in vitro* transwell (Boyden chamber) migration assay. Data demonstrate statistically significant migration of hMSC-CD146^+^ in 10 % fetal bovine serum (FBS) as compared with hMSC-CD146^−^ under the same conditions. gray box, 0.2 % FBS hMSC-CD146^+/−^; white box, 10 % FBS hMSC-CD146^+^; black box, 10 % FBS hMSC-CD146, n = 2 independent experiments. *P < 0.05. **b** IVIS^®^ *in vivo* imaging of hMSC-CD146^+^ and hMSC-CD146^−^ cells at days 0, 3, and 6 after intravenous tail vein injection. As can be observed in the images and from the radiance plots, hMSC-CD146^+^ cells (n = 4 mice) demonstrate migration away from the lung area at days 3 and 6, and hMSC-CD146^−^ cells (n = 5 mice) display less migration in comparison with hMSC-CD146^+^

To explore other biological characteristics associated with the CD146 status, we examined the cells’ ability for chemotaxis *in vitro* and *in vivo*. The hMSC-CD146^+^ sorted population exhibited enhanced chemotactic responses to 10 % FBS when compared with the 0.2 % FBS control in an *in vitro* transwell migration assay. In contrast, hMSC-CD146^−^ cells demonstrated no significant migration between 0.2 % (control) and 10 % FBS (Fig. 5a). To examine the *in vivo* relevance of this observation, we injected LUC2-labelled hMSC-CD146^+^ and hMSC-CD146^–^ into the tail vein of immune-deficient mice with prior closed femur fracture. The mice were examined at d0, d3, and d6 (Fig. 5b). In all mice injected with hMSC-CD146^–^, the cells migrated to the lung, where the majority remained throughout the 6-day period. In contrast, hMSC-CD146^+^ cells were observed to migrate away from the lung area starting at day 3 and were recruited to both the injured right leg and non-injured left leg. By day 6, hMSC-CD146^+^ exhibited wider systemic distribution throughout the animals. Analysis of the bioluminescence radiance captured by using IVIS imaging demonstrated statistically significant differences between the lung and lower body radiance. Data from the lung radiance demonstrated a shift from significantly higher levels of hMSC-CD146^+^ radiance at d0 (*P* < 0.05) to significantly lower radiance at day 6 (*P* < 0.01) as compared with hMSC-CD146^−^. Analysis of the lower body demonstrated significantly increased radiance in the hMSC-CD146^+^ at all time points (*P < 0.05, **P < 0.01) as compared with hMSC-CD146^−^ radiance.

## Discussion

Identification of prospective markers defining the functional ability of hMSCs is an important pre-requisite for use of hMSCs in therapy. In the present study, we employed CD146 to functionally phenotype BM hMSCs. Our results demonstrate that CD146 defines a population with bone-forming capacity as well as ability for *in vivo* trans-endothelial migration and homing to injured bone sites.

Previous studies have suggested a number of biological roles for CD146, including trans-endothelial migration [24–27], cell proliferation regulation [27–29], and a role in cancer metastases [30–32]. In addition, CD146 has also been proposed as a pericyte cell marker [33, 34] and as a cell marker for hMSCs and osteoblastic cells [27, 33, 35]. Here, we report that CD146 status is associated with progenitor functions of hMSCs which include differentiation into osteoblasts and adipocytes as well as ability for trans-endothelial migrations *in vivo*.

Both hMSC-CD146^+^ and hMSC-CD146^−^ cells were able to differentiate readily into osteoblastic and adipocytic cells with similar efficiency, and both populations formed heterotopic bone and BM when implanted in immune-deficient mice. Our results corroborate previous results that also demonstrated, in primary BM hMSCs, that CD146 status did not influence response to differentiation induction to osteoblasts, adipocytes, or chondrocytes [28]. Recently, Gothard et al. compared CD146^+^ cells with Stro1^+^ and demonstrated similar gene expression profiles [13]. Similarly, Russell et al. reported that CD146^+^ cells are enriched in multipotent hMSCs [7]. Although these studies did conduct side-by-side comparison of CD146^+^ and CD146^−^ hMSC populations, the current data suggest that CD146^+^ status defines the progenitor cell population of hMSCs.

Morphological changes of the cytoskeleton, including reorganization of actin fibres, are important for osteoblast cell differentiation [36]. Recent data have demonstrated an increase in the density of F-actin during hMSC differentiation [37]. Using the SER features from the Harmony software, we analyzed the cytoplasmic texture and demonstrated significant differences in the sizes of the F-actin fibres between hMSC-CD146^+^ and hMSC-CD146^−^ cells, and actin fibres were significantly larger in the hMSC-CD146^−^ population. Sonowal et al. [38], McBeath et al. [39], and Treiser et al. [40] have reported an association between actin fibre and cytoskeletal modification with osteoblastic differentiation. Thus, the observed changes in actin fibres in CD146^−^ cells may contribute to their more mature OB phenotype.

We identified that CD146 defines cells with ability for trans-endothelial migration. Statistically significant data demonstrated that hMSC-CD146^+^ cells migrated away from the lungs; however, homing to a site of injury was not consistently observed. Consistent with these findings, we observed that the hMSC-CD146^+^ cells exhibited a smaller cell size and cytoskeletal morphology, suggesting enhanced motility. Our results corroborate results from previous investigators that CD146 confers additional functional phenotype on hMSCs. Espangolle et al. reported that CD146^+^ cells exhibited an enriched vascular smooth muscle cell phenotype [28]. Ye et al. [26] demonstrated that CD146 acts as a receptor for Wnt5A, mediating induced cell migration. Along similar lines to our study, Blocki et al. [33] reported that although both CD146^+^ and CD146^−^ populations of BM hMSCs exhibited an equivalent differentiation ability for adipogenic and osteoblastic lineages, only CD146^+^ cells formed endothelial tubular networks on Matrigel™. The endothelial-like phenotype may be related to trans-endothelial migration ability.

We identified a significant difference between the amount of bone formed by hMSC-CD146^+^ and hMSC-CD146^−^ cells. hMSC-CD146^−^ formed more bone but demonstrated a 10-fold decrease in BM compared with hMSC-CD146^+^. hMSC-CD146^−^ maintained their CD146^−^ status throughout the experiments, but hMSC-CD146^+^ cells were observed to be returning to a phenotypic equilibrium after six passages post-cell sort. Thus, it is possible that utilization of cells where 100 % were positive for CD146 may enhance the quantity of BM and decrease the amount of bone formed. Previous reports have demonstrated that CD146^+^ hMSCs are located in the perivascular spaces [14] and have been associated with re-establishment of the haematopoietic microenvironment [18]. In comparison, CD146^−^ cells were found to line the bone cavity [14]. The different locations of CD146^+^ or CD146^−^ cells could suggest that, within the heterogeneous MSC population, cells have different degrees of maturity: CD146^−^ form more bone and less marrow and are thus more mature, whereas CD146^+^ retain more plasticity (that is, in addition to bone formation and BM formation, they have a trans-endothelial migration capacity).

## Conclusions

We hypothesize that CD146^+^ cells are recruited to bone surfaces by trans-endothelial migration [41]. Further maturation to active osteoblasts is associated with loss of CD146 and thus cells located on active bone-forming surfaces are CD146^−^. Changes in the expression of CD146 reveal a dual function in hMSC biology: a role in enhancing migration and recruitment to bone surfaces (CD146^+^) and a role in osteoblastic commitment and bone formation (CD146^−^). Further studies examining molecular details of mechanisms underlying these two functions need to be investigated.

## Additional files

Additional file 1: Table S1. Sequence data for primers used within these studies. (DOCX 13 kb) Additional file 2: Figure S1. Characterization of hMSC-LUC2 as compared with the parental cell line hMSC-TERT. **A** Morphology. **B** Long-term growth rate. **P* < 0.05. **C** Flow cytometric analysis of CD marker expression. Scale bar = 500 or 100 μm. *hMSC* human mesenchymal stem cell. (TIF 423 kb) Additional file 3: Figure S2. Characterization of osteoblast and adipocyte differentiation capacity of hMSC-TERT and hMSC-LUC2. **A** Osteoblastic differentiation. **B** Adipocytic differentiation. **P* < 0.05, ***P* < 0.005, ****P* < 0.001; scale bar = 100 μm: black box, hMSC-TERT; white box, hMSC-LUC2. *ALP* alkaline phosphatase, *AZR* Alizarin red, *hMSC* human mesenchymal stem cell. (TIF 522 kb) Additional file 4: Figure S3. Characterization and comparison of *in vivo* bone formation of hMSC-TERT and hMSC-LUC2. **A, B** Scanned images of hMSC-TERT and hMSC-LUC2 implants. Hematoxylin-and-eosin staining of hMSC-TERT **(C)** and hMSC-LUC2 **(D). E** hMSC-LUC2 implants: human-specific vimentin staining demonstrating the human origin of the cells. Images of chondrocyte **(F)**, blood vessel **(G)**, and osteoclast **(H)**. Scale bar = 100 μm. *hMSC* human mesenchymal stem cell. (TIF 1294 kb) Additional file 5: Figure S4.
*In vitro* adipocytic differentiation of hMSC-CD146^+^ and hMSC-CD146^−^ cell populations. **A** Oil red O staining. **B** Reverse transcription-polymerase chain reaction analysis of adipocytic gene expression. n = 3 independent experiments, mean ± standard error of the mean; white box, hMSC-CD146^+^; black box, hMSC-CD146^−^; scale bar = 100 μm. *hMSC* human mesenchymal stem cell. (TIF 505 kb) Additional file 6: Figure S5. CD profile of hMSC-TERT, hMSC-CD146^+^, and hMSC-CD146^−^ populations prior to *in vivo* implantation. *hMSC* human mesenchymal stem cell. (TIF 150 kb) Additional file 7: Figure S6. Scanned images (hematoxylin and eosin and human-specific vimentin) of whole implants of hMSC-CD146^+^ and hMSC-CD146^−^ cells implanted into immune-compromised mice for 8 weeks. Cells of murine origin can be observed in the non-stained areas of implants as demonstrated by *arrows. hMSC* human mesenchymal stem cell. (TIF 1385 kb) Additional file 8: Figure S7. Validation of human-specific vimentin staining. **A, B** Human mesenchymal stem cells (hMSCs) implanted on hydroxyapatite tricalcium phosphate/Triosite (HA/TCP) in immune-compromised mice. **C, D** Murine mesenchymal stem cells (mMSCs) implanted on HA/TCP in immune-compromised mice. No vimentin staining can be observed in murine cells. Scale bar = 100 μm. (TIF 1979 kb)

## Abbreviations

ALP: Alkaline phosphatase
BM: Bone marrow
BSA: Bovine serum albumin
CD146: Cluster of differentiation 146
CT: Cycle threshold
EDTA: Ethylenediaminetetraacetic acid
FACS: Fluorescence-activated cell sorting
FBS: Fetal bovine serum
HA/TCP: Hydroxyapatite tricalcium phosphate/Triosite
hMSC: Human skeletal (stromal) mesenchymal stem cell
lgCD146: Long form of CD146
LUC2: Luciferase
MSC: Mesenchymal stem cell
ORO: Oil red O
PBS: Phosphate-buffered saline
PCR: Polymerase chain reaction
RT-PCR: Reverse transcription-polymerase chain reaction
SER: Saddles, edges, ridges
shCD146: Short form of CD146
