# Supplementary material for: CD146/MCAM defines functionality of human bone marrow stromal stem cell populations
Source: Stem Cell Res Ther. 2016 Jan 11;7:4. doi: 10.1186/s13287-015-0266-z (PMC4710006; doi:10.1186/s13287-015-0266-z)
Supplement: Additional file 1: Table S1. — Sequence data for primers used within these studies. (DOCX 13 kb) [file 13287_2015_266_MOESM1_ESM.docx]

| **Gene** | **Forward primer** | **Reverse primer** | **Product size** |
| --- | --- | --- | --- |
| bActin | ATTGGCAATGAGCGGTTCCG | AGGGCAGTGATCTCCTTCTG | 211 |
| Runx2 | TGGTTACTGTCATGGCGGGTA | TCTCAGATCGTTGAACCTTGCTA | 101 |
| COL1a1 | AGGGCTCCAACGAGATCGAGATCCG | TACAGGAAGCAGACAGGGCCAACGTCG | 223 |
| ALPL | ACGTGGCTAAGAATGTCATC | CTGGTAGGCGATGTCCTTA | 476 |
| SPARC | AACGAAGAAAGCGAAGCAGAAGTG | CTGACCATCATAGCCATCGTAGCCT | 450 |
| BGLAP | CATGAGAGCCCTCACA | AGAGCGACACCCTAGAC | 310 |
| OPN | CCA AGT AAG TCC AAC GAA AG | GGT GAT GTC CTC GTC TGT A | 347 |
| BGN | CCTCCCCTCTCCAGGTCCAT | CTTCTGCAGCTTCCGCAGTG | 451 |
| ELN | TGTCCATCCTCCACCCCTCT | CTCCTGGGACACCACCAAGC | 300 |
| DCN | CGCCTCATCTGAGGGAGCTT | GGACCGGGTTGCTGAAAAGA | 201 |
| CD146 | CCAAGGCAACCTCAGCCATGTC | CTCGACTCCACAGTCTGGGACGACT | 438 |
| shCD146* | GCCAGTCCTCATACCAGAGC | TCTCTCCATCTCCTGCTTCC | 192 |
| lgCD146* | TGGTTTGTACACCTTGCAGAGTATTC | TGGGCAGCCGGTAGTTGA | 95 |
| aP2 | GCCAGGAATTTGACGAAGTC | TGG TTG ATT TTC CAT CCC AT | 107 |
| ADNPQ | TGTTGCTGGGAGCTGTTCTACTG | ATGTCTCCCTTAGGACCAATAAG | 234 |
| LPL | GAGATTTCTCTGTATGGCACC | CTGCAAATGAGACACTTTCTC | 275 |
| C/EBPα | CACGAAGCACGATCAGTCC | CATTGCACAAGGCACTGC | 126 |
| PPARγ2 | TTCTCCTAT TGACCCAGAAAGC | CTCCACTTTGATTGCACTTTGG | 307 |

*Kebir et al 2010

Table S1: primers used for gene expression
