# Supplementary figures and images for: CD146/MCAM defines functionality of human bone marrow stromal stem cell populations
Source: Stem Cell Res Ther. 2016 Jan 11;7:4. doi: 10.1186/s13287-015-0266-z (PMC4710006; doi:10.1186/s13287-015-0266-z)

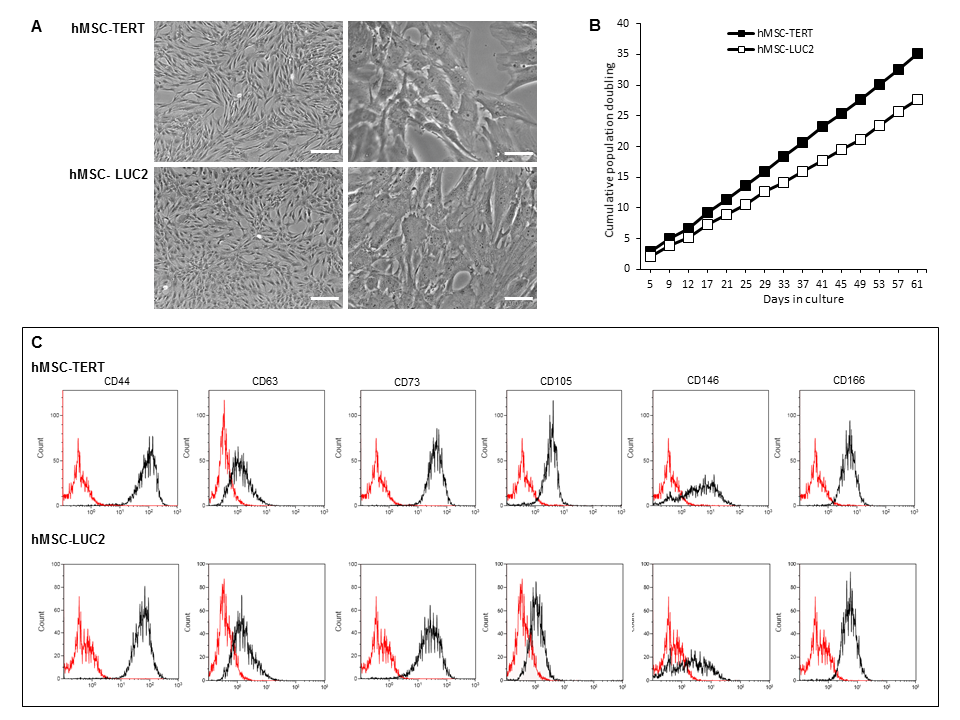

Supplement: Additional file 2: Figure S1. — Characterization of hMSC-LUC2 as compared with the parental cell line hMSC-TERT. A Morphology. B Long-term growth rate. *P < 0.05. C Flow cytometric analysis of CD marker expression. Scale bar = 500 or 100 μm. hMSC human mesenchymal stem cell. (TIF 423 kb) [file 13287_2015_266_MOESM2_ESM.tif]

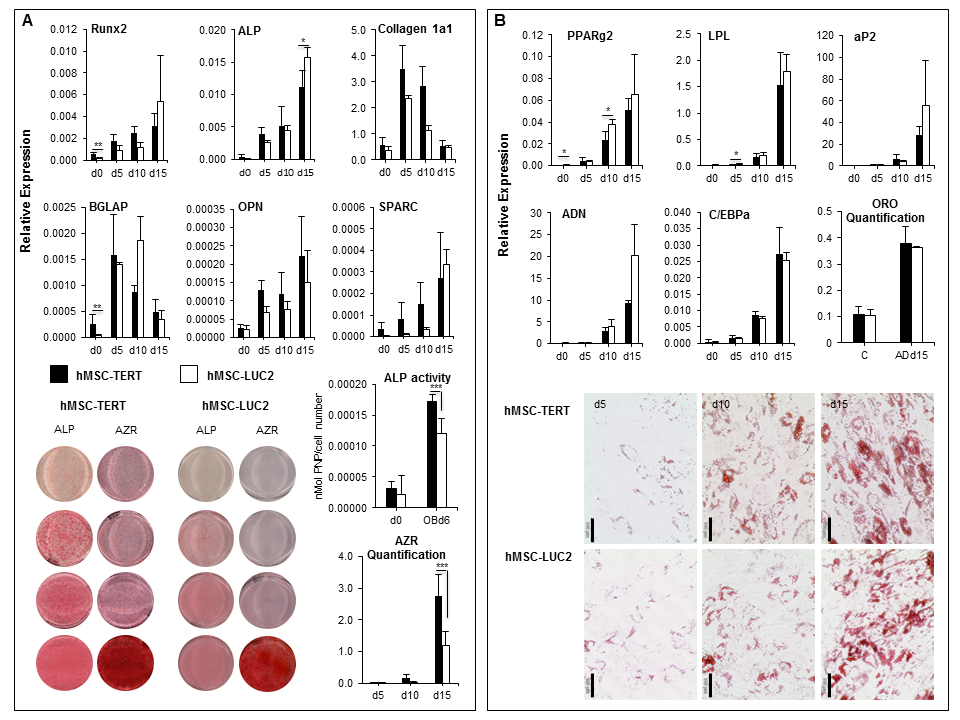

Supplement: Additional file 3: Figure S2. — Characterization of osteoblast and adipocyte differentiation capacity of hMSC-TERT and hMSC-LUC2. A Osteoblastic differentiation. B Adipocytic differentiation. *P < 0.05, **P < 0.005, ***P < 0.001; scale bar = 100 μm: black box, hMSC-TERT; white box, hMSC-LUC2. ALP alkaline phosphatase, AZR Alizarin red, hMSC human mesenchymal stem cell. (TIF 522 kb) [file 13287_2015_266_MOESM3_ESM.tif]

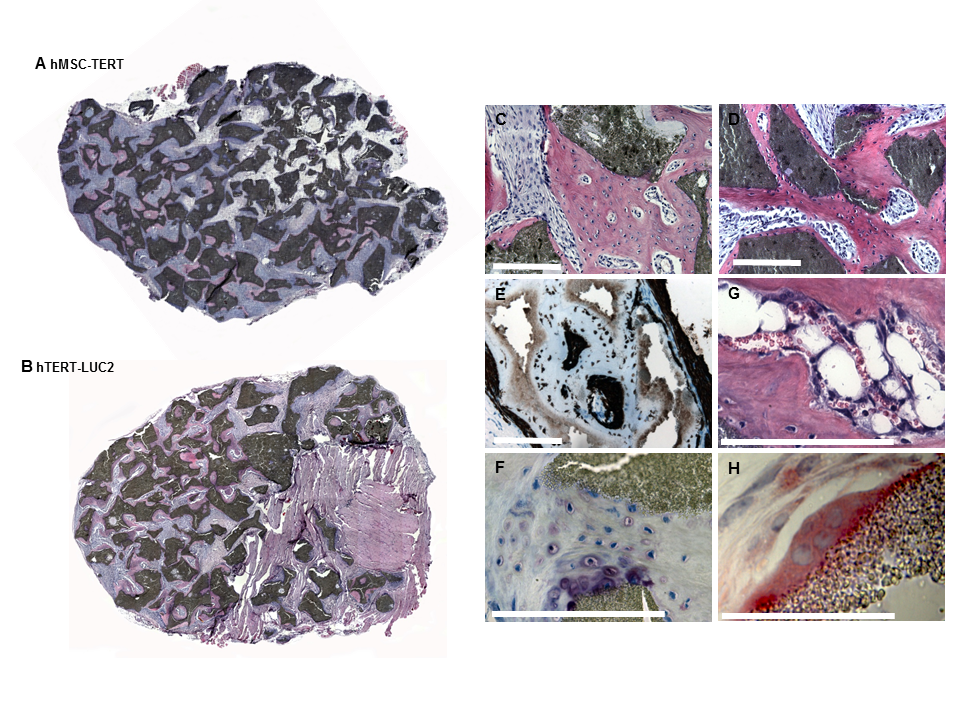

Supplement: Additional file 4: Figure S3. — Characterization and comparison of in vivo bone formation of hMSC-TERT and hMSC-LUC2. A, B Scanned images of hMSC-TERT and hMSC-LUC2 implants. Hematoxylin-and-eosin staining of hMSC-TERT (C) and hMSC-LUC2 (D). E hMSC-LUC2 implants: human-specific vimentin staining demonstrating the human origin of the cells. Images of chondrocyte (F), blood vessel (G), and osteoclast (H). Scale bar = 100 μm. hMSC human mesenchymal stem cell. (TIF 1294 kb) [file 13287_2015_266_MOESM4_ESM.tif]

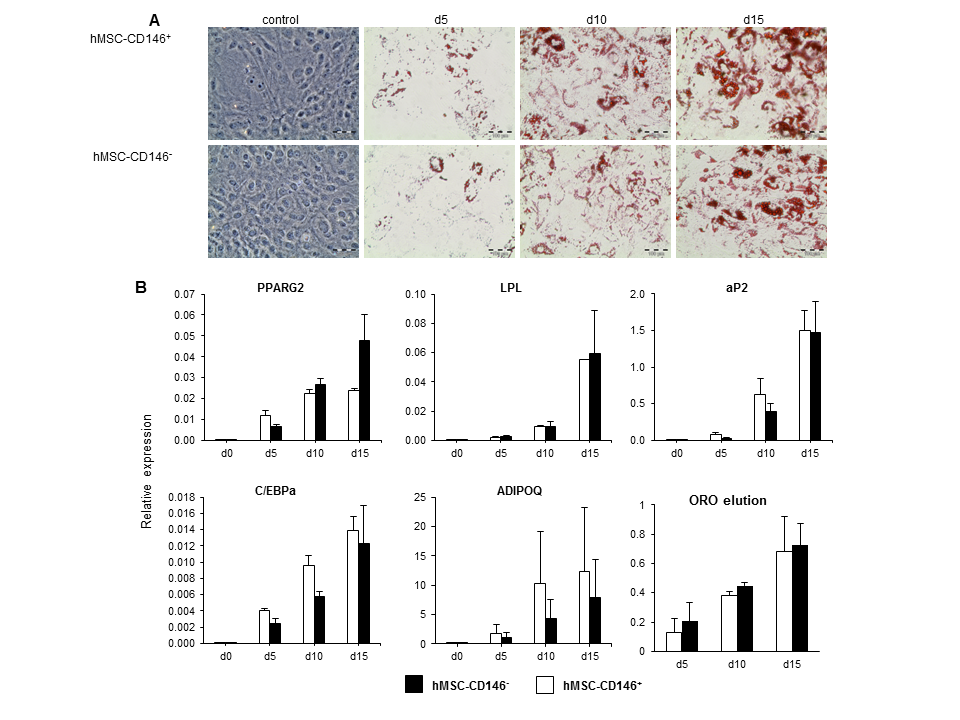

Supplement: Additional file 5: Figure S4. — In vitro adipocytic differentiation of hMSC-CD146+ and hMSC-CD146− cell populations. A Oil red O staining. B Reverse transcription-polymerase chain reaction analysis of adipocytic gene expression. n = 3 independent experiments, mean ± standard error of the mean; white box, hMSC-CD146+; black box, hMSC-CD146−; scale bar = 100 μm. hMSC human mesenchymal stem cell. (TIF 505 kb) [file 13287_2015_266_MOESM5_ESM.tif]

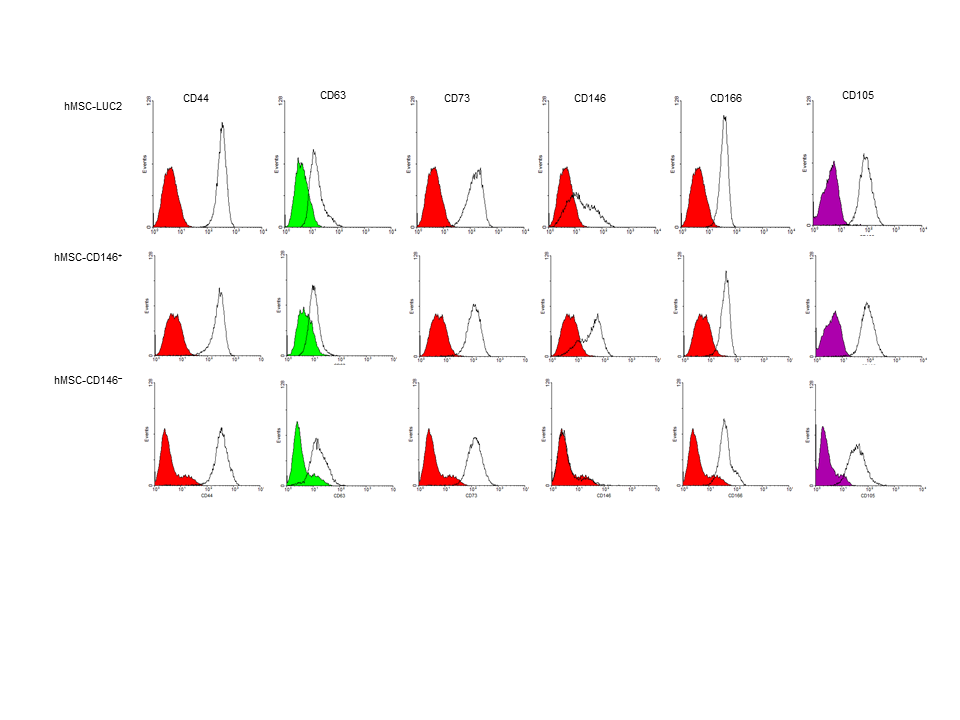

Supplement: Additional file 6: Figure S5. — CD profile of hMSC-TERT, hMSC-CD146+, and hMSC-CD146− populations prior to in vivo implantation. hMSC human mesenchymal stem cell. (TIF 150 kb) [file 13287_2015_266_MOESM6_ESM.tif]

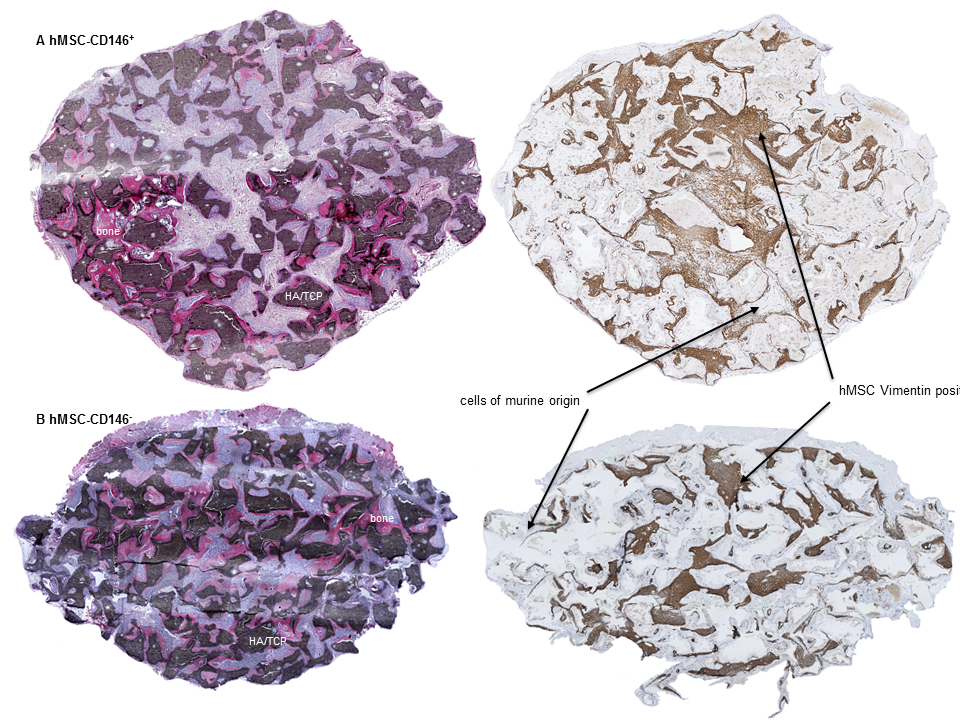

Supplement: Additional file 7: Figure S6. — Scanned images (hematoxylin and eosin and human-specific vimentin) of whole implants of hMSC-CD146+ and hMSC-CD146− cells implanted into immune-compromised mice for 8 weeks. Cells of murine origin can be observed in the non-stained areas of implants as demonstrated by arrows. hMSC human mesenchymal stem cell. (TIF 1385 kb) [file 13287_2015_266_MOESM7_ESM.tif]

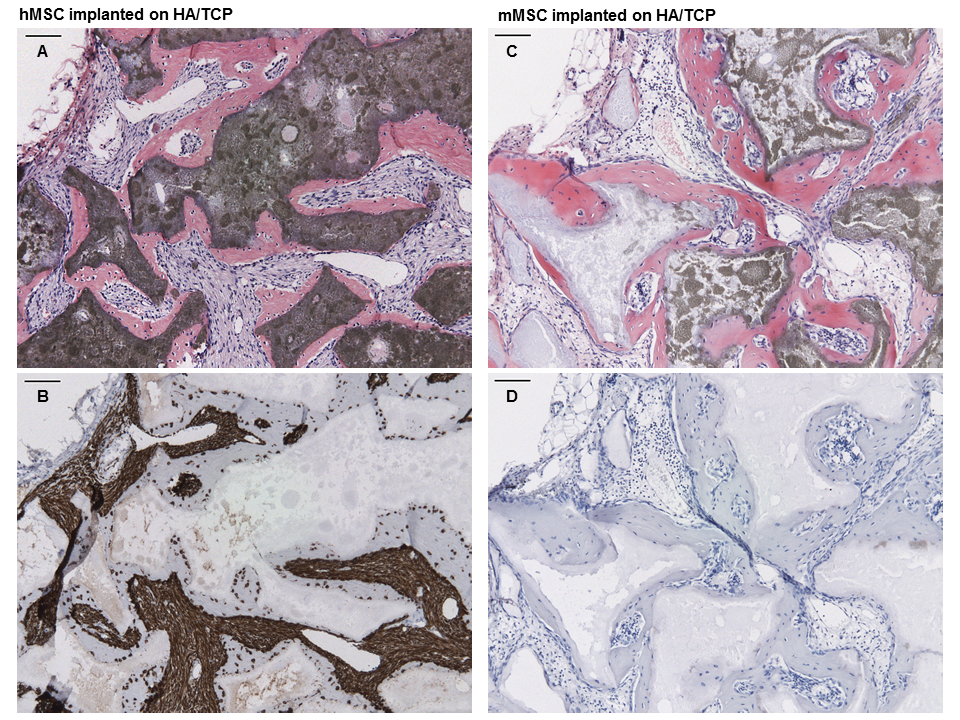

Supplement: Additional file 8: Figure S7. — Validation of human-specific vimentin staining. A, B Human mesenchymal stem cells (hMSCs) implanted on hydroxyapatite tricalcium phosphate/Triosite (HA/TCP) in immune-compromised mice. C, D Murine mesenchymal stem cells (mMSCs) implanted on HA/TCP in immune-compromised mice. No vimentin staining can be observed in murine cells. Scale bar = 100 μm. (TIF 1979 kb) [file 13287_2015_266_MOESM8_ESM.tif]
